# Childhood infections, orchitis and testicular germ cell tumours: a report from the STEED study and a meta-analysis of existing data

**DOI:** 10.1038/bjc.2012.45

**Published:** 2012-02-16

**Authors:** B Trabert, B I Graubard, R L Erickson, K A McGlynn

**Affiliations:** 1 Hormonal and Reproductive Epidemiology Branch, Division of Cancer Epidemiology and Genetics, National Cancer Institute, NIH, DHHS, 6120 Executive Boulevard, Suite 550, Rockville, MD 20852-7234, USA; 2 Biostatistics Branch, Division of Cancer Epidemiology and Genetics, National Cancer Institute, NIH, DHHS, 6120 Executive Boulevard, Rockville, MD 20852-7234, USA; 3 Walter Reed Army Institute of Research, 503 Robert Grant Avenue, Silver Spring, MD 20910-7500, USA

**Keywords:** testicular germ cell tumours, childhood infections, mumps, orchitis, case–control

## Abstract

**Background:**

Similarities between the age-specific incidence pattern of testicular germ cell tumours (TGCTs) and the age-specific incidence pattern of cancers of viral origin prompted us to evaluate the relationship between common infections occurring during childhood or young adult life and TGCT using existing data from the US Servicemen's Testicular Tumor Environmental and Endocrine Determinants (STEED) case–control study.

**Methods:**

TGCT cases diagnosed between 2002 and 2005 (*n*=767) were matched on age, race and serum draw date to at least one control (*n*=929).

**Results:**

None of the infections evaluated were associated with TGCT risk. Further, a meta-analysis of mumps and mumps orchitis or orchitis infection did not support an association with TGCT (mumps pooled odds ratio (OR): 1.03, 95% confidence interval (CI): 0.89–1.20; mumps orchitis or orchitis pooled OR: 1.80, 95% CI: 0.74–4.42).

**Conclusion:**

Based on our evaluation of childhood and early life infections and meta-analyses of mumps and mumps orchitis and/or orchitis, TGCT does not appear to be associated with common childhood infections.

Testicular germ cell tumours (TGCTs) are the most common cancer among young men in many countries (Chia *et al*, 2010). There are few established risk factors beyond age, race/ethnicity, adult height, history of cryptorchidism and family history of TGCT (McGlynn and Cook, 2009). The great majority of TGCTs arise in young men between the ages of 15 and 40 years. Although there has been much interest in a possible perinatal aetiology of these young adult tumours, non-perinatal factors may also have a role. In addition, the age-specific incidence pattern of TGCT resembles that of some cancers with viral aetiology, such as young-adult non-Hodgkin's lymphoma (NHL) (Newell *et al*, 1984; Algood *et al*, 1988).

Mumps, a viral disease, is manifested by inflammation of the salivary glands (Plotkin and Rubin, 2008). In the absence of vaccination, most persons will be infected by this disease in young adulthood (Plotkin and Rubin, 2008) and up to 37% of post-pubertal males will develop orchitis. A number of studies support an association between post-pubertal mumps and/or orchitis and TGCT (Mills *et al*, 1984; Brown *et al*, 1987; Swerdlow *et al*, 1987), whereas evidence linking TGCT and childhood mumps is largely null (Henderson *et al*, 1979; Loughlin *et al*, 1980; Coldman *et al*, 1982; Moss *et al*, 1986; Brown *et al*, 1987; Haughey *et al*, 1989; Stone *et al*, 1991; UK Testicular Cancer Study Group, 1994; Petridou *et al*, 1997).

Using existing data from the US Servicemen's Testicular Tumor Environmental and Endocrine Determinants (STEED) study, we evaluated the association between common childhood infections and TGCT. In addition, using published reports in the literature, we conducted a meta-analysis of mumps and/or orchitis and risk of TGCT.

## Materials and methods

### Study population

Participants in the STEED study were enrolled between 2002 and 2005 (McGlynn *et al*, 2007). Briefly, men between 18 and 45 years of age who had at least one serum sample stored in the US Department of Defense Serum Repository (DoDSR, Silver Spring, MD, USA) were eligible for the study. Men who developed TGCT while on active duty were eligible to participate as cases, whereas men who did not develop TGCT were eligible to participate as controls. TGCT diagnoses were limited to classic seminoma or nonseminoma (embryonal carcinoma, yolk sac carcinoma, choriocarcinoma, teratoma and mixed germ cell tumour). Eligible controls (*n*=928) were individually matched to cases (*n*=767) based on age at diagnosis (within 1 year), ethnicity (white, black and other) and date when serum was donated (within 30 days). Informed consent was obtained from all the participants. The study was approved by the institutional review boards of the National Cancer Institute (Bethesda, MD, USA) and the Walter Reed Army Institute of Research (Silver Spring, MD, USA).

The study questionnaire elicited information on risk factors for TGCT, as well as self-reported history of common childhood infections and age at onset (mumps, chicken pox including varicella or shingle, measles, roseola or Sixth disease and mononucleosis). The questionnaire also ascertained a self-reported history of orchitis, inflammation and/or swelling of the testicles prior to the reference date and the age at onset of orchitis. It also included a self-reported history of urinary tract infections, inflammation in the groyne area and sexually transmitted infections (chlamydia, genital herpes, gonorrhoea and syphilis).

### Statistical analysis

Odds ratios (ORs) and 95% confidence intervals (CIs) were calculated using unconditional logistic regression adjusting for matching factors and TGCT risk factors (cryptorchidism, family history of testicular cancer and adult height). Sensitivity analyses were conducted, whereby study participants with self-reported age at infection within 1 year of the reference age were excluded. Analyses were conducted of all TGCT together and for seminoma and nonseminoma using SAS 9.1 software (SAS Institute Inc., Cary, NC, USA). All tests were two-sided, with *P*-value <0.05 considered statistically significant.

### Meta-analysis

PubMed (http://www.ncbi.nlm.nih.gov/) was searched in a systematic manner for all observational studies of mumps, orchitis or mumps/orchitis and TGCT through 1 August 2011, using the following keywords: TGCT, testicular cancer, mumps, mumps orchitis AND orchitis, or their combinations. The references of all publications were searched for additional studies, and the PubMed option ‘related articles’ was used to search for potentially relevant articles.

Meta-analyses using fixed-effect methods and DerSimonian and Laird random-effects methods (DerSimonian and Laird, 1986) were performed in STATA v.11.2 (StataCorp., College Station, TX, USA). Summary ORs were compared and the *I*^*2*^ value and its 95% uncertainty intervals were used to assess the consistency of the study-specific estimates (Higgins *et al*, 2003). Publication bias was evaluated using a funnel plot of the log OR against the s.e. of the log OR. Small-study effects were tested using the Egger and Begg's tests and sensitivity analyses were conducted, whereby each study was omitted in turn and the summary estimate recalculated.

## Results

The distributions of selected characteristics of the study population are provided in Table 1. Cases were more likely than controls to report a history of cryptorchidism (adjusted OR: 3.31; 95% CI: 1.84–5.97).

### Mumps and orchitis

There was no association between history of mumps without orchitis and TGCT (OR: 0.98; 95% CI: 0.76–1.27) (Table 2). Overall history of orchitis was associated with an increased risk of TGCT (OR: 2.38; 95% CI: 1.56–3.63), however, this association was limited to orchitis diagnosed within one calendar year of the reference date (OR: 23.16; 95% CI: 5.53–96.99). Orchitis diagnosed more than 1 year prior to the reference age was not associated with risk (OR: 1.17; 95% CI: 0.71–1.94).

We further evaluated mumps and orchitis occurring around puberty (⩾10 years of age) as the testes undergo maturation and peripubertal infection accompanied with inflammation may be an aetiologically relevant event. Orchitis at ⩾10 years of age (OR: 1.12; 95% CI: 0.67–1.90) and mumps infection at ⩾10 years of age were not associated with TGCT (OR: 1.34; 95% CI: 0.71–2.51) (Supplementary Table 1).

### Common childhood infections, genital conditions and genital tract infections

History of measles, chicken pox, roseola/sixth disease and mononucleosis were not associated with TGCT overall or by histological type (Supplementary Table 2). Similarly, self-reported inflammation in the groyne area was not associated (OR: 1.20; 95% CI: 0.83–1.74), nor were there patterns suggesting that urinary tract infection or sexually transmitted infections were associated.

With the exception of results presented above for orchitis infection where the age at diagnosis of orchitis was within 1 year of reference age, the ORs for association between infection and TGCT remained unchanged in sensitivity analyses excluding participants with self-reported age at infection within 1 year of the reference age.

### Meta-analysis of the association between mumps, orchitis and TGCT

**Figure 1:**
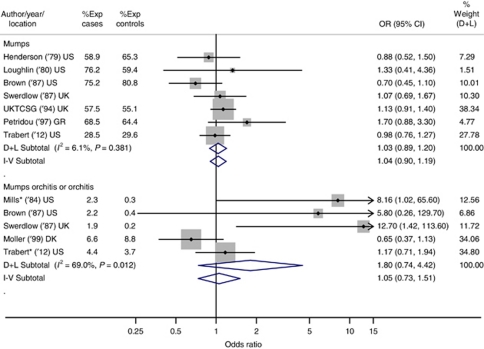
Summary odds ratios (ORs) and 95% confidence intervals (CIs) for the association between mumps (top), mumps orchitis or orchitis (bottom) and TGCT. Summary ORs and 95% CIs are reported based on fixed-effect (I–V) and random-effects (D+L) meta-analytic models. All statistical tests were two-sided. ‘% Weight’ describes the weighting each study contributes to the random-effects summary OR. The dot on each square represents the study-specific OR (also listed in the ‘OR (95% CI)’ column), and the size of the shaded square is illustrative of the random-effects study weighting. The horizontal lines represent the 95% CIs; the lines ending in an arrow indicate that this interval transcends the region plotted. The diamonds represent the summary ORs and 95% CIs for the mumps group (first subtotal), the mumps orchitis or orchitis group (second subtotal) and overall (last). For the ‘mumps orchitis or orchitis’ plot, studies denoted with an ‘^*^’ included orchitis cases only. Abbreviations: DK=Denmark; GR=Greece; UK=United Kingdom; UKTCSG=United Kingdom Testicular Cancer Study Group; US=United States; Yr=year study published.

The pooled summary OR based on the random-effects model for mumps was 1.03 (95% CI: 0.89–1.20); suggesting no association with TGCT (Figure 1). The consistent fixed- and random-effects pooled ORs and *I*^*2*^ of 6.1% indicate very little heterogeneity among the six studies (*P*-value: 0.38).

For mumps orchitis or orchitis, the random-effects model yielded a pooled OR of 1.80 (95% CI: 0.74–4.42). The disparate fixed- and random-effects pooled ORs and *I*^*2*^ of 69.0% (*P*-value=0.01) suggested considerable heterogeneity across study-specific ORs for the five studies. Baujat's plot indicated that the two recent studies contributed most to the heterogeneity (Moller and Skakkebaek, 1999 and the current study). When these studies were removed, the *I*^*2*^ value dropped to 0.0% (*P*-value: 0.91) and the pooled ORs for fixed- and random-effects models became identical (OR (95% CI): 9.06 (2.3–35.2)) and indicative of an increased risk of TGCT.

## Discussion

History of orchitis, mumps and other childhood infections were not risk factors for TGCT in this study.

As summarised in the meta-analysis of mumps orchitis or orchitis, increased risk of TGCT was observed in three prior studies (Mills *et al*, 1984; Brown *et al*, 1987; Swerdlow *et al*, 1987) and a null association was observed in the current study and another recent study conducted by Moller and Skakkebaek (1999). These earlier studies, however, are based on small sample sizes (*n*=8, 6 and 5 exposed cases, respectively), whereas the latter studies included 32 and 34 exposed cases, respectively. Not included in the meta-analysis were three studies that did not provide case/control counts or risk estimates, two of which reported an increased risk with orchitis (Beard *et al*, 1977) and one reported a null association (Stone *et al*, 1991).

Common childhood infections, inflammation in the groyne area, history of urinary tract infection and sexually transmitted infections were not associated with TGCT. The lack of association with childhood infectious diseases, including mumps, was consistent with most published reports (Henderson *et al*, 1979; Loughlin *et al*, 1980; Coldman *et al*, 1982; Moss *et al*, 1986; Brown *et al*, 1987; Haughey *et al*, 1989; Stone *et al*, 1991; UK Testicular Cancer Study Group, 1994; Petridou *et al*, 1997). Our meta-analysis of mumps included six studies in addition to the current study (Henderson *et al*, 1979; Loughlin *et al*, 1980; Brown *et al*, 1987; Swerdlow *et al*, 1987; UK Testicular Cancer Study Group, 1994; Petridou *et al*, 1997); all of these studies reported a null association. Four additional studies also reported a null association, however, these studies were not included in the meta-analysis because of the lack of case/control counts or risk estimates (Coldman *et al*, 1982; Moss *et al*, 1986; Haughey *et al*, 1989; Stone *et al*, 1991). Most authors reported, as we do, a lack of association between sexually transmitted infections and TGCT (Coldman *et al*, 1982; Moss *et al*, 1986; Brown *et al*, 1987; Swerdlow *et al*, 1987).

Strengths of the current study include the large sample size, high response proportion (91% of cases and 81% of controls), and that cases and controls were drawn from the same well-defined population (McGlynn *et al*, 2007). Further, the study included only pathologically confirmed TGCT. As with all case–control studies, however, limitations include the reliance on participant recall, which may be more selective among cases. We attempted to address this issue with sensitivity analyses that excluded self-reported infection that occurred in the calendar year prior to the reference date.

As hypothesised in the introduction, we evaluated infectious agents and risk of TGCT based on similarities in age-specific incidence patterns for TGCT and cancers with infectious etiologies. Specifically, NHL also occurs in young men, and studies have shown that some cases are the result of an infectious agent following the paralytic polio model. Given the epidemiologic similarities between TGCT and NHL, Newell *et al* (1984) hypothesised that the paralytic polio model may be relevant to TGCT aetiology (Newell *et al*, 1984). However, our data suggest that, unlike NHL, TGCT does not appear to be associated with common childhood infections.

## Figures and Tables

**Table 1:** Characteristics of study participants stratified by case-control status and TGCT histology, STEED Study, 2002–2005

	**Controls (*n*=928)**		**All TGCT (*n*=767)**		**Seminoma (*n*=324)**		**Nonseminoma (*n*=442)**	
	***n***	**%^a^**	***n***	**%^a^**	***n***	**%^a^**	***n***	**%^a^**
*Age (years)*								
<25	318	34.3	278	36.2	61	18.8	216	48.9
25–29	277	29.8	217	28.3	97	29.9	120	27.1
30–34	174	18.8	137	17.9	78	24.1	59	13.3
35–39	120	12.9	101	13.2	64	19.8	37	8.4
40+	39	4.2	34	4.4	24	7.4	10	2.3
								
*Race*								
White	788	84.9	647	84.4	260	80.2	387	87.6
Black	35	3.8	22	2.9	12	3.7	10	2.3
Other	105	11.3	98	12.8	52	16.0	45	10.2
								
*Reference height (cm)*								
<172.73	260	15.3	157	9.3	62	3.7	95	5.6
172.73–177.80	257	15.2	223	13.2	98	5.8	125	7.4
177.81–182.88	240	14.2	197	11.6	87	5.1	110	6.5
>182.88	171	10.1	188	11.1	77	4.5	110	6.5
								
*Cryptorchidism*								
Yes	16	1.7	41	5.3	11	3.4	30	6.8
No	912	98.3	726	94.7	313	96.6	412	93.2
								
*First- or second-degree family history of testicular cancer*								
Yes	14	1.5	33	4.3	17	5.2	16	3.6
No	915	98.5	734	95.7	307	94.8	426	96.4

Abbreviations: STEED=Servicemen's Testicular Tumor Environmental and Endocrine Determinants; TGCT=testicular germ cell tumour.

a Percents may not sum to 100 because of missing values.

**Table 2:** Association of mumps, orchitis and testicular germ cell tumours according to histology: STEED Study 2002–2005

	**Controls**	**All Cases**			**Seminoma**			**Nonseminoma**		
	***n* (%)**	***n* (%)**	**OR^a^**	**(95% CI)**	***n* (%)**	**OR^a^**	**(95% CI)**	***n* (%)**	**OR^a^**	**(95% CI)**
*Mumps*										
Yes	256 (27.6)	194 (25.3)	0.98	(0.76, 1.27)	103 (31.8)	0.89	(0.63, 1.25)	91 (20.6)	1.04	(0.75, 1.44)
No	610 (65.7)	486 (63.4)	1.00	Reference	184 (56.8)	1.00	Reference	302 (68.3)	1.00	Reference
										
Orchitis										
Yes	36 (3.9)	68 (8.9)	2.38	(1.56, 3.63)	22 (6.8)	1.52	(0.86, 2.71)	46 (10.4)	3.09	(1.93, 4.93)
No	889 (95.7)	692 (90.2)	1.00	Reference	300 (92.6)	1.00	Reference	391 (88.5)	1.00	Reference
										
*Orchitis stratified by age at infection with respect to reference age*										
Within 1 year	2 (< 0.1)	36 (4.7)	23.16	(5.53, 96.99)	13 (4.0)	19.99	(4.3, 92.73)	23 (5.2)	23.58	(5.45, 101.99)
>1 year	34 (3.7)	32 (4.4)	1.17	(0.71, 1.94)	9 (2.8)	0.60	(0.28, 1.32)	23 (5.2)	1.72	(0.98, 3.03)
No	889 (95.7)	692 (90.2)	1.00	Reference	300 (92.6)	1.00	Reference	391 (88.5)	1.00	Reference

Abbreviations: CI=confidence interval; OR=odds ratio; STEED=Servicemen's Testicular Tumor Environmental and Endocrine Determinants.

a Adjusted for matching factors, cryptorchidism, adult height and family history of testicular cancer.
