# Supplementary material for: Childhood infections, orchitis and testicular germ cell tumours: a report from the STEED study and a meta-analysis of existing data
Source: Br J Cancer. 2012 Feb 16;106(7):1331–4. doi: 10.1038/bjc.2012.45 (PMC3314781; doi:10.1038/bjc.2012.45)
Supplement: Supplementary Tables 1 and 2 [file bjc201245x1.doc]

| Supplemental Table 1. Association of mumps or orchitis diagnosed at age 10 or older and testicular germ cell tumors according to histology: STEED Study 2002-2005. | | | | | | | | | | |
| --- | --- | --- | --- | --- | --- | --- | --- | --- | --- | --- |
|  | Controls | All Cases | | | Seminomas | | | Nonseminomas | | |
|  | n (%) | n (%) | OR | (95% CI) | n (%) | OR | (95% CI) | n (%) | OR | (95% CI) |
| Mumps diagnosed at age 10 or older | | | |  |  |  |  |  |  |  |
| Yes | 19 (2.1) | 19 (2.5) | 1.34 | (0.71, 2.51) | 11 (3.4) | 1.14 | (0.51, 2.53) | 8 (1.8) | 1.41 | (0.64, 3.08) |
| No | 610 (65.7) | 486 (63.4) | 1.00 | reference | 184 (56.8) | 1.00 | reference | 302 (93.2) | 1.00 | reference |
| Orchitis diagnosed at age 10 or older† | | |  |  |  |  |  |  |  |  |
| Yes | 34 (3.7) | 63 (8.2) | 2.40 | (1.56, 3.71) | 21 (6.5) | 1.62 | (0.91, 2.90) | 42 (9.5) | 3.07 | (1.90, 4.98) |
| No | 889 (95.7) | 692 (90.2) | 1.00 | reference | 300 (92.6) | 1.00 | reference | 391 (88.0) | 1.00 | reference |
| *Adjusted for matching factors, cryptorchidism, adult height, and family history of testicular cancer.  STEED=Servicenmen’s Testicular Tumor Environmental and Endocrine Determinants  †Analyses for orchitis excluded participants that reported orchitis infection within one calendar year of reference date.  OR=Odds Ratio  CI=Confidence Interval | | | | | | | |  |  |  |

| Supplemental Table 2. Association of childhood infections, genital conditions and genital tract infections and testicular germ cell tumors according to histology: STEED Study 2002-2005. | | | | | | | | | | |
| --- | --- | --- | --- | --- | --- | --- | --- | --- | --- | --- |
|  | Controls | All Cases | | | Seminomas | | | Nonseminomas | | |
|  | n (%) | n (%) | OR | (95% CI) | n (%) | OR | (95% CI) | n (%) | OR | (95% CI) |
| Measles |  |  |  |  |  |  |  |  |  |  |
| Yes | 211 (22.7) | 161 (21.0) | 0.99 | (0.75, 1.30) | 81 (25.0) | 0.77 | (0.54, 1.10) | 80 (18.1) | 1.16 | (0.83, 1.62) |
| No | 638 (68.7) | 512 (66.8) | 1.00 | reference | 198 (61.1) | 1.00 | reference | 314 (71.0) | 1.00 | reference |
| Chicken Pox, Varicella, or Shingles | | |  |  |  |  |  |  |  |  |
| Yes | 821 (88.4) | 650 (84.8) | 0.86 | (0.62, 1.20) | 270 (83.3) | 0.84 | (0.55, 1.28) | 380 (86.0) | 0.88 | (0.59, 1.33) |
| No | 86 (9.3) | 76 (9.9) | 1.00 | reference | 36 (11.1) | 1.00 | reference | 40 (9.1) | 1.00 | reference |
| Roseola or Sixth Disease | | |  |  |  |  |  |  |  |  |
| Yes | 4 (0.4) | 6 (0.8) | 1.64 | (0.45, 5.99) | 4 (1.2) | 2.96 | (0.68, 12.77) | 2 (0.5) | 0.89 | (0.14, 5.75) |
| No | 913 (98.3) | 741 (96.6) | 1.00 | reference | 314 (96.9) | 1.00 | reference | 427 (96.6) | 1.00 | reference |
| Mononucleosis |  |  |  |  |  |  |  |  |  |  |
| Yes | 101 (10.9) | 89 (11.6) | 1.10 | (0.81, 1.50) | 41 (12.7) | 1.18 | (0.79, 1.77) | 48 (10.9) | 1.00 | (0.69, 1.46) |
| No | 822 (88.5) | 658 (85.8) | 1.00 | reference | 277 (85.5) | 1.00 | reference | 381 (86.2) | 1.00 | reference |
| Inflammation in Groin Area | |  |  |  |  |  |  |  |  |  |
| Yes | 64 (6.9) | 62 (8.1) | 1.20 | (0.83, 1.74) | 28 (8.6) | 1.08 | (0.66, 1.75) | 34 (7.7) | 1.25 | (0.80, 1.96) |
| No | 863 (92.9) | 699 (91.1) | 1.00 | reference | 294 (90.7) | 1.00 | reference | 404 (91.4) | 1.00 | reference |
| Urinary tract infection | |  |  |  |  |  |  |  |  |  |
| Yes | 96 (10.3) | 75 (9.8) | 0.96 | (0.69, 1.33) | 39 (12.0) | 0.93 | (0.61, 1.42) | 36 (8.1) | 0.96 | (0.63, 1.45) |
| No | 831 (89.5) | 690 (90.0) | 1.00 | reference | 285 (88.0) | 1.00 | reference | 405 (91.6) | 1.00 | reference |
| Chlamydia |  |  |  |  |  |  |  |  |  |  |
| Yes | 41 (4.4) | 29 (3.8) | 0.84 | (0.51, 1.37) | 11 (3.4) | 0.64 | (0.31, 1.29) | 18 (4.1) | 1.00 | (0.56, 1.79) |
| No | 886 (95.4) | 736 (96.0) | 1.00 | reference | 313 (96.6) | 1.00 | reference | 423 (95.7) | 1.00 | reference |
| Genital herpes |  |  |  |  |  |  |  |  |  |  |
| Yes | 14 (1.5) | 15 (2.0) | 1.47 | (0.69, 3.1) | 9 (2.8) | 1.46 | (0.6, 3.55) | 6 (1.4) | 1.48 | (0.55, 4.02) |
| No | 913 (98.3) | 750 (97.8) | 1.00 | reference | 315 (97.2) | 1.00 | reference | 435 (98.4) | 1.00 | reference |
| Gonorrhea |  |  |  |  |  |  |  |  |  |  |
| Yes | 43 (4.6) | 34 (4.4) | 1.04 | (0.65, 1.68) | 18 (5.6) | 0.97 | (0.54, 1.76) | 16 (3.6) | 1.08 | (0.58, 1.99) |
| No | 884 (95.2) | 731 (95.3) | 1.00 | reference | 306 (94.4) | 1.00 | reference | 425 (96.2) | 1.00 | reference |
| Syphilis |  |  |  |  |  |  |  |  |  |  |
| Yes | 3 (0.3) | 6 (0.8) | 2.47 | (0.61, 10.08) | 3 (0.9) | 1.40 | (0.27, 7.26) | 3 (0.7) | 4.43 | (0.85, 22.98) |
| No | 924 (99.5) | 759 (99.0) | 1.00 | reference | 321 (99.1) | 1.00 | reference | 438 (99.1) | 1.00 | reference |
| *Adjusted for matching factors, cryptorchidism, adult height, and family history of testicular cancer.  STEED=Servicenmen’s Testicular Tumor Environmental and Endocrine Determinants  OR=Odds Ratio  CI=Confidence Interval | | | | | | | | | | |
